# Co-treatment with arsenic trioxide and ganciclovir reduces tumor volume in a murine xenograft model of nasopharyngeal carcinoma

**DOI:** 10.1186/1743-422X-10-152

**Published:** 2013-05-16

**Authors:** Mark D Sides, Meredith L Sosulski, Fayong Luo, Zhen Lin, Erik K Flemington, Joseph A Lasky

**Affiliations:** 1 Department of Medicine, Tulane University School of Medicine, New Orleans, LA, USA; 2 Department of Pathology, Tulane University School of Medicine, New Orleans, LA, USA; 3 Department of Pulmonary Medicine and Critical Care – SL9, Tulane University School of Medicine, 1430 Tulane Ave, New Orleans, LA 70112, USA

**Keywords:** Nasopharyngeal carcinoma, Epstein-Barr virus, Arsenic trioxide, Ganciclovir, Promyelocytic leukemia nuclear bodies, Xenograft, CNE1

## Abstract

We have previously shown that disruption of promyelocytic leukemia nuclear bodies (PML NBs) is sufficient to activate the EBV lytic cycle thus making infected cells susceptible to ganciclovir (GCV) mediated killing *in vitro*. Here we show that co-administration of GCV and arsenic trioxide (ATO), a PML NB disruptor, reduces tumor volume in a xenograft model of nasopharyngeal carcinoma utilizing CNE1 cells. When administered at pharmacologic levels, both GCV and ATO reduced tumor growth while co-treatment with GCV + ATO resulted in a diminution of tumor volume. Treatment with GCV or ATO individually resulted in an increased number of apoptotic cells while co-treatment with GCV + ATO synergistically induced apoptosis. Treatment with ATO or co-treatment with GCV + ATO resulted in expression of EBV lytic proteins. These data suggest that co-treatment with GCV + ATO may provide an effective treatment for nasopharyngeal carcinoma patients.

## Background

Nasopharyngeal carcinoma (NPC) has a high occurrence rate in endemic areas of Southern China, Southeast Asia and North Africa and comprises a substantial health burden [1-3]. Additionally, a majority of new cases occur in countries with limited resources for current therapies or early detection of NPC [4]. Most cases in endemic areas consist of WHO Type III anaplastic NPC with a high correlation of Epstein Barr virus (EBV) positivity while the majority of cases in non-endemic areas consist of keratinizing squamous cell carcinomas [5-9]. The genetic and geographic pattern to NPC occurrence rates indicates multifocal etiology in that those of Cantonese descent born in North America display occurrence rates greater than their geographical counterparts, but less than that in endemic areas [10-15].

Current treatment for NPC consists of radiotherapy and chemotherapeutics with early detection associated with better clinical outcomes [2,3]. Accordingly, in more developed areas where early detection and standard treatments are readily available, mortality rates are declining [16]. In rural areas, initial presentation tends to be late stage with few treatment options [17]. The deliberate induction of EBV lytic reactivation to actuate antiviral susceptibility has been proposed as a potential therapeutic for EBV associated malignancies [18-25]. Induction of EBV lytic protein expression, specifically the virally encoded protein kinase, is essential for activation of the anti-herpesviral prodrugs [26]. Recently, we have shown that reactivation of EBV lytic protein expression by arsenic trioxide (ATO) treatment confers susceptibility to the antiviral drug ganciclovir (GCV) *in vitro*[27]. Here we investigated the efficiency of the ATO mediated reactivation strategy in combination with the antiviral ganciclovir (GCV) to target EBV positive cancer cells *in vivo* in a tumor xenograft model. Treatment with ATO alone reduced tumor growth consistent with previous reports [28-30]. Co-treatment with GCV and ATO (CoTx) demonstrated an advantage over ATO alone, reducing tumor volume to below baseline.

## Results

### Co-treatment with GCV and ATO reduces tumor volume *in vivo*

**Figure 1:**
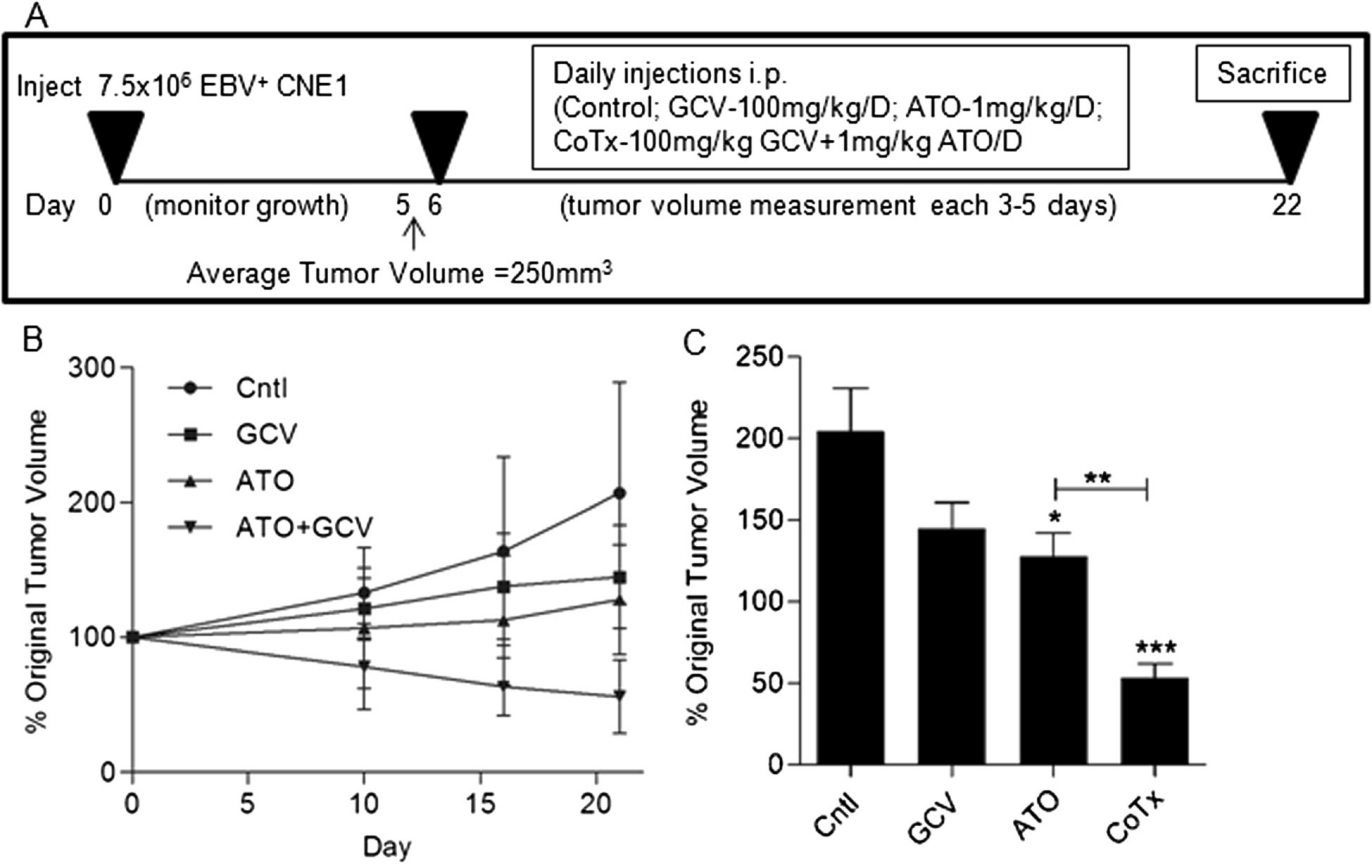
**Co-treatment with GCV + ATO reduces tumor volume in a xenograft model of NPC A) Overview of tumor xenograft model and treatment regimen. B**) Growth curve of the average tumor volume of each treatment group. Tumor volumes were measured by a blind investigator and compared to starting volumes as determined by the L*W^2^/2 formula. **C**) Comparative ending tumor volume as a percentage of baseline measurement. (*) denotes p < 0.05; (**) denoted p < 0.01; (***) denotes p < 0.005.

We have previously reported the ability of ATO treatment to induce ganciclovir susceptibility in EBV positive NPC cells *in vitro*[27]. To investigate whether this treatment would translate to an *in vivo* model of NPC, the EBV positive nasopharyngeal carcinoma cell line, CNE1 < BX1 > cells were inoculated subcutaneously into NU/NU mice. Daily intraperitoneal injections of the indicated treatment were given starting 5 days post inoculation and animals were sacrificed at day 22 (Figure 1A). Tumors in control animals displayed an accelerating growth curve over the 21 days while treatment with either GCV or ATO alone inhibited tumor growth relative to control (Figure 1B). Co-treatment (CoTx) with GCV plus ATO reduced the tumor volume to below the baseline (approximately 50% of original tumor volume, Figure 1C). The difference in tumor volume in CoTx animals was statistically significant from all groups. Though the tumor volume difference between ATO treated and control was statistically significant, the tumor volume difference between GCV treated and either control or ATO was not statistically significant.

### CNE1 < BX1 > cells display EBER positivity within the sections

**Figure 2:**
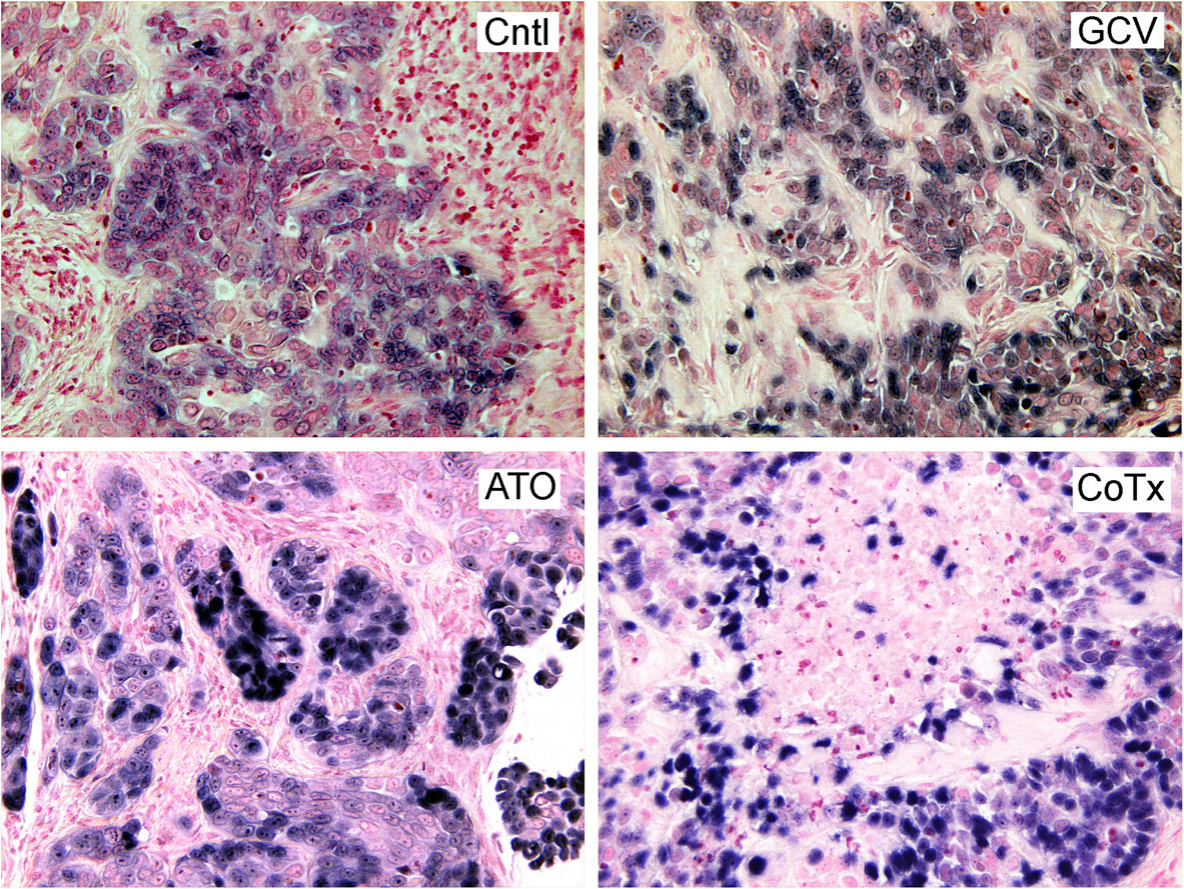
**CNE1 < BX1 > cells within the tumors display EBER positivity and are morphologically distinct from mouse cell infiltrates.** In sections of tumor explants 22 days post injection, EBER-1 and EBER-2 were detected by cytogenic *in situ* hybridization and stained with NBT/BCIP (blue). Nuclei were stained with Nuclear Fast Red counterstain (original magnification of 200×).

Loss of the EBV genomes in NPC cells passaged in immunodeficient mice has been reported [31]. Chromogenic *in situ* hybridization staining for EBER-1 and EBER-2 positive cells was performed to establish EBV positivity and to illustrate the morphological differences between the CNE1 < BX1 > cells and possible infiltration of murine cells into the tumor (Figure 2). CNE1 < BX1 > cells displayed EBER positivity in all sections, appear morphologically distinct in size and shape, and were easily distinguishable from infiltrating murine cells. In sections from ATO and CoTx tumors, CNE1 < BX1 > cells appear in pockets surrounded by murine cells to a greater extent than in control or GCV treated sections.

### Treatment with ATO or co-treatment with GCV + ATO increases apoptosis

**Figure 3:**
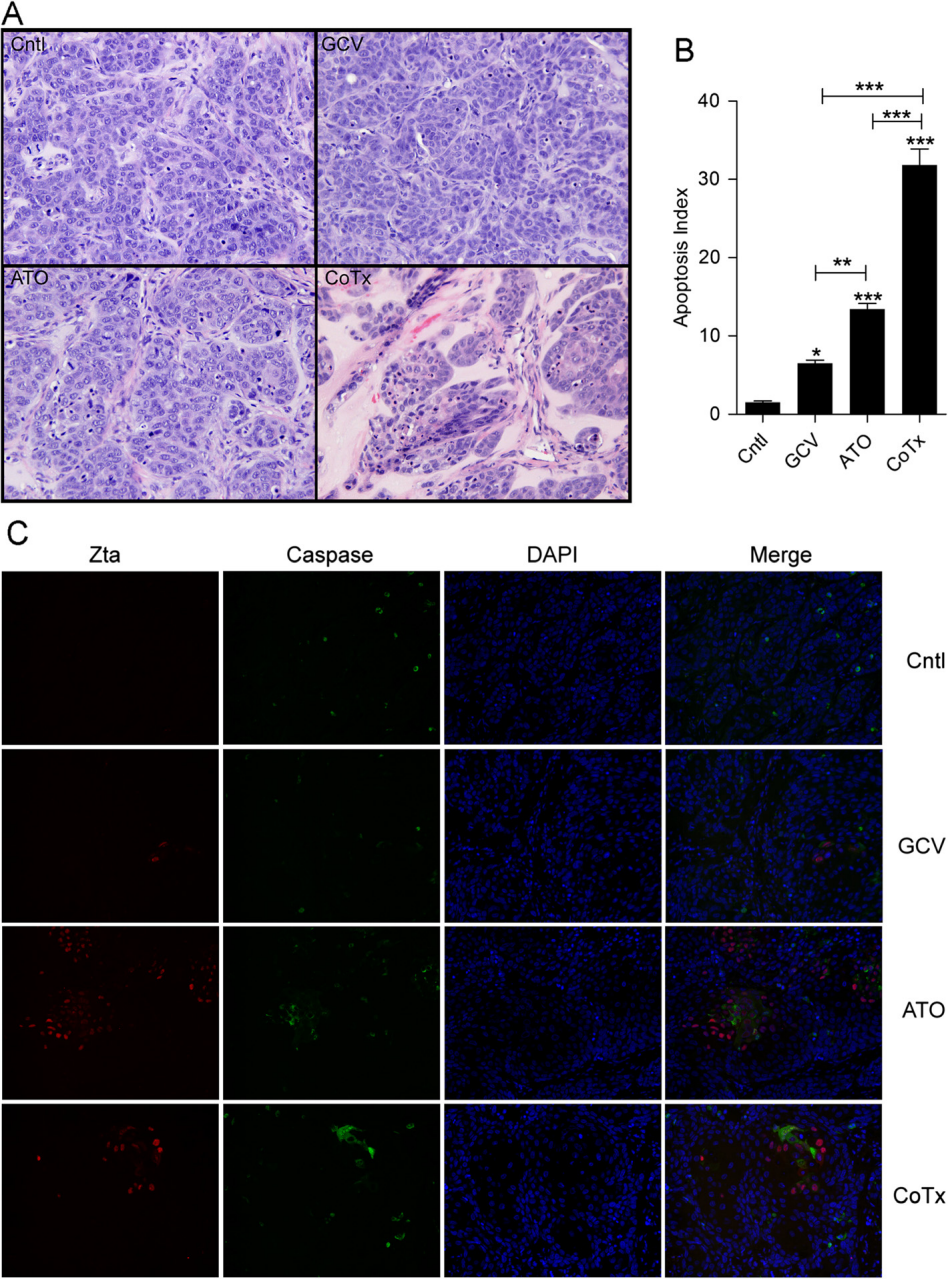
**Co-treatment with GCV + ATO increases apoptosis within tumor sections. A**) Representative fields of H&E stained sections from tumor explants 22 days post injection (original magnification of 200×). **B**) Total CNE1 < BX1 > nuclei and those displaying apoptotic changes were counted in 10 random fields from each group comprising multiple sections. **C**) Tumor xenograft sections from each of the treatment groups were stained for the immediate early EBV protein Zta (red) and activate Caspase-3 (green). Nuclei were stained with DAPI (blue) (original magnification of 200X).

The previously demonstrated increase in apoptosis when CNE1 < BX1 > cells were treated with GCV + ATO *in vitro*[27] together with the observed reduction in tumor volume measured in the CoTx group for this manuscript suggests that the decrease in tumor volume *in vivo* occurs through apoptosis. To assess the extent of apoptosis or change in tumor architecture, tumor sections were fixed and stained with H & E (Figure 3A). ATO and CoTx sections showed a reduced cellular density compared with control or GCV sections. Additionally ATO and CoTx sections displayed an increased number of apoptotic bodies compared to control. To quantify this increase, cells displaying apoptotic nuclear morphology were counted in 10 fields from several section samples (Figure 3B). GCV and ATO treatment sections displayed an increased apoptotic index (AI) when compared to control, and CoTx sections displayed an increased AI when compared to control, GCV or ATO sections.

Active caspase-3 was assessed by immunofluorescence microscopy and samples were co-stained for expression of the EBV encoded immediate early lytic protein Zta to further quantify the extent of apoptosis (Figure 3C). Expression of active caspase-3 correlated with nuclei displaying apoptotic changes in all sections. Sections from ATO and CoTx samples showed an increase in active caspase-3 as well as an increase in the number of Zta positive cells. Zta expression in ATO and CoTx sections displayed a distinct pattern of pockets of positivity characterized by lower cellular density and active caspase-3 expression. This pattern was present to a lesser extent in GCV sections.

### Treatment with ATO or co-treatment with GCV + ATO reduces PML NB fluorescent intensity and activates expression of EBV lytic proteins

**Figure 4:**
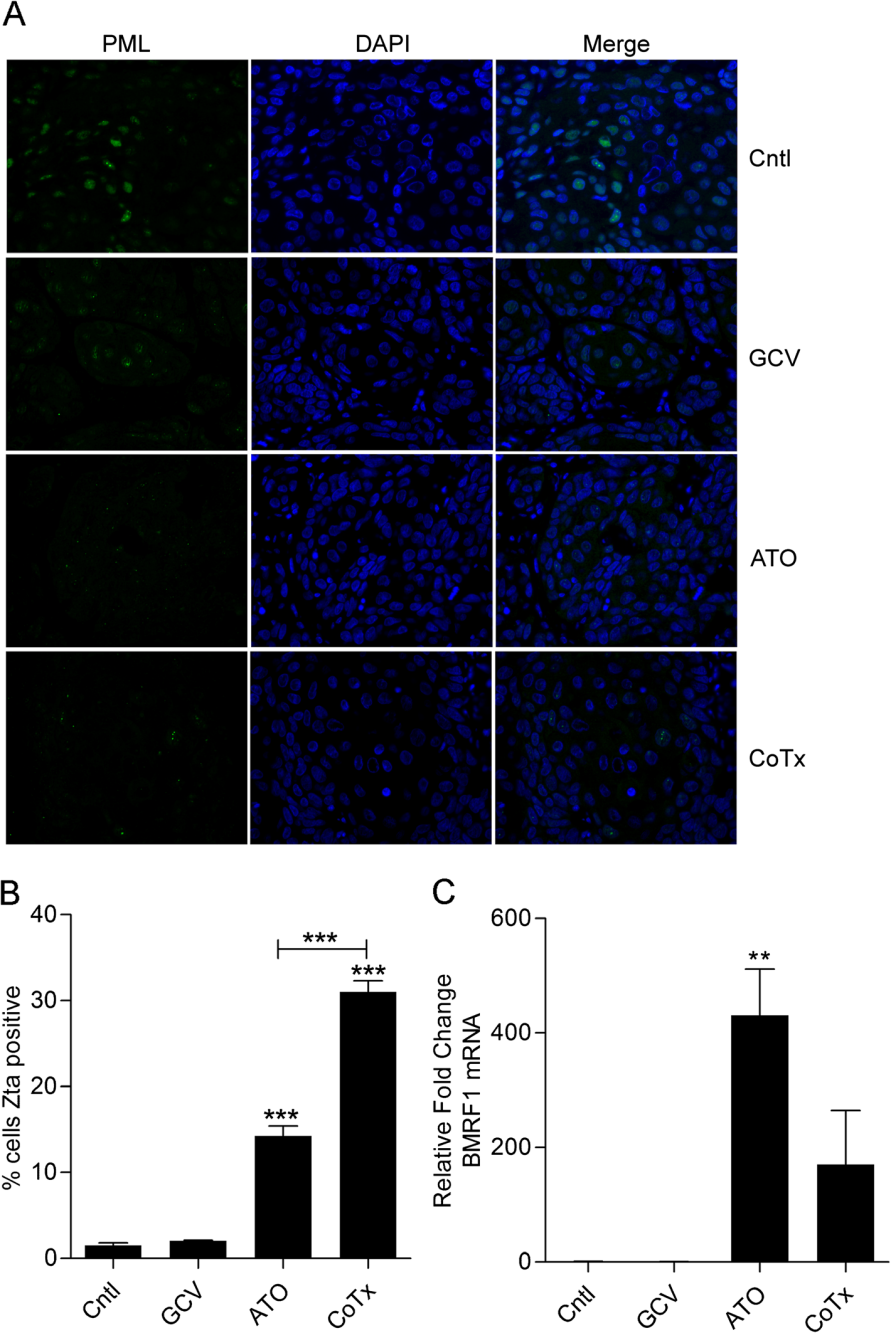
**ATO and CoTx disruption of PML NBs induces EBV lytic protein expression. A**) Sections from tumor explants from each of the 4 treatment groups were stained for PML (green). Nuclei were stained with DAPI (blue) (original magnification of 400X). **B**) Zta positive nuclei and total CNE1 < BX1 > nuclei were counted in random fields comprising multiple sections from each treatment group (Figure 3C). **C**) RNA was isolated from tumor explant tissue samples and levels of the EBV early protein BMRF1 gene product were assessed by reverse transcriptase real-time PCR.

ATO has previously been shown to disrupt PML NBs and increase expression of EBV lytic proteins in EBV positive NPC cells [27]. To assess whether PML NBs were affected *in vivo* by ATO or GCV + ATO treatment, PML NBs were assessed by immunofluorescence microscopy (Figure 4A). In control samples, PML expression showed distinct punctate nuclear staining. Though PML NBs were detected in both the ATO or CoTx sections, the immunofluorescence signal of PML NBs was greatly reduced when compared to control sections. Treatment with ATO alone or CoTx resulted in an increase in the number of cells expressing the EBV immediate early protein Zta (Figure 3C). To quantify this increase, the number of Zta positive cells and total CNE1 < BX1 > cells were counted in 10 random fields from each sample and the percentage of Zta positive cells was graphed (Figure 4B). In ATO sections, 14% of cells showed Zta positivity while 31% of cells in CoTx section were Zta positive, which correlates with our earlier *in vitro* findings [27]. Levels of the DNA processivity factor BMRF1, a Zta responsive early protein, was assessed by quantitative real-time reverse transcriptase PCR (Figure 4C) in order to affirm the expression of downstream EBV lytic genes. RNA from the ATO and CoTX tumors showed increased BMRF1 expression.

## Discussion

ATO has been utilized alone and in conjunction with radiotherapy in both *in vitro* and *in vivo* models of NPC [28-30,32-35]. In studies utilizing tumor xenograft models of NPC, treatment with ATO alone produced a decrease in tumor growth compared to controls at doses of 5–10 mg/kg/d. The use of GCV in a previous study utilizing NPC tumor xenograft models produced only slight growth inhibition [36]. Previous work from our laboratory has shown enhancement of EBV lytic protein expression in EBV positive NPC cells *in vitro* through disruption of PML NBs in response to low dose (give dose) ATO and increased apoptosis with the addition of GCV [27].

In the current study, treatment with GCV alone slowed tumor growth (though not statistically significant) suggesting a level of spontaneous reactivation in the CNE1 < BX1 > cell line. This is consistent with the presence of a low number of Zta positive cells in control sections and the low number of pockets of Zta positive cells with a similar pattern of active caspase-3 detection. Treatment with low dose ATO (1 mg/kg/d) showed growth inhibition compared to control, consistent with previous reports utilizing higher doses. Importantly, the combination of GCV and ATO reduced tumor volume compared to baseline measurements, and histology of these tumors displayed lower cellular density and an increased number of Zta positive and apoptotic cells compared to either control or single treatment samples. During the course of experiment, no negative effects were observed with any of the treatments and weight gain was similar among all groups.

Recently, Wildman et al. reported stable disease management and improved quality of life utilizing GCV in conjunction with valproic acid as a reactivating agent in 3 patients with end-stage NPC, thus supporting a reactivation strategy as a viable treatment in EBV positive refractory patients [37]. Feng and Kenney utilized a murine xenograft model and demonstrated that valproic acid in conjunction with gemcitabine significantly inhibited tumor growth over the course of treatment [21], but not a reduction in tumor volume as we report here using a similar model with GCV + ATO co-treatment. The notable differences between the studies preclude a direct comparison of the results. Notably, lymphoblastoid cell derived tumors may react differently than NPC cell derived tumors and unlike GCV, gemcitabine induced cell death is independent of EBV lytic reactivation. The current report builds on these pioneering studies [21,37] and provides important evidence that ATO in conjunction with GCV may be a feasible and effective treatment for NPC. The utilization of readily available portable infusion therapies that are well tolerated and demonstrate limited side effects would be especially useful in developing countries where the majority of new NPC cases arise.

## Materials and methods

### Tumor xenograft

CNE1 < BX1 > cells have been previously described and were cultured in Dulbecco’s Modified Eagle Medium plus 10% fetal bovine serum with 700 ug/ml G418 (Gibco/Life Technologies, Grand Island, NY) [27]. Animal experiments were performed in accordance with Tulane University Institutional Animal Care and Use Committee approved protocols and standards. Six to eight week old outbred NU/NU mice (strain code 088) weighing 18–20 grams were obtained from Charles River Laboratories (Wilmington, MA) and housed in sterile conditions. CNE1 < BX1 > cells (7.5×10^6^ cells/mouse) were trypsinized and mixed with growth factor reduced matrigel (BD Bioscience, Franklin Lakes, New Jersey) and injected subcutaneously on the right flank of each mouse. Tumor growth was assessed for 5 days at which time the average tumor volume was 250 mm^3^. Animals were randomly assigned to control (n = 6), GCV (100 mg/kg/d, n = 6), ATO (1 mg/kg/d, n = 7), or CoTx (100 mg/kg/d GCV + 1 mg/kg/d ATO, n = 7). Working solutions were diluted such that injection volumes for each group were equal. Tumor volume was assessed at 3–5 day intervals by a blinded investigator using the L*W^2^/2 formula. At 22 days post injection, mice were euthanized and tumors were harvested for paraffin sectioning and mRNA isolation. Isolation of mRNA and quantitative real time reverse transcription PCR detection of BMRF1 mRNA levels was done as previously described with the following exceptions [27]. At harvest, ½ of the tumor volume was flash frozen in liquid nitrogen and stored at −80 deg C. Samples were homogenized utilizing homogenized utilizing a rotor-stator homogenizer in RLT Plus buffer. Data is representative of repeated experiments.

### Histology and apoptotic Index (AI) analysis

Formalin preserved tumor samples were paraffin imbedded, sectioned on slides and H&E stained by the Histology Core, Tulane Center for Stem Cell Research and Regenerative Medicine. Deparaffinized and rehydrated sections were bioled 5 minutes in 2xSSC and bathed 10 minutes in 50 mM ammonium chloride prior to blocking and application of antibodies. Primary antibodies to Zta (1:100) (Argene, New York), LMP1 (1:200), PML (1:500) (Santa Cruz Biotechnology, Santa Cruz, CA), and cleaved Caspase-3 (1:200) (Cell Signaling, Danvers MA) with secondary antibodies Alexa Fluor 594 goat anti-mouse (1:500) and Alexa Fluor 488 goat anti-rabbit (1:500) antibodies (Invitrogen) were used in immunofluorescence detection. An apoptotic index (AI) was assessed from H & E stained slides. Light microscopy images were taken from ten random fields with each slide averaging between 200 (CoTx) and 700 (Cntl) CNE1 < BX1 > cells per image. The AI was calculated as the ratio of cells with apoptotic bodies to the total number of CNE1 < BX1 > nuclei per field.

### Chromogenic *in situ* hybridization (CISH)

Chromogenic *in situ* hybridization detection of EBV encoded EBERs was performed by the Molecular Pathology Laboratory, Department of Pathology and Laboratory Medicine using the ZytoFast © EBV-CISH System. Briefly, biotin conjugated probes specific to EBER-1 and EBER-2 were allowed to hybridize followed by complexing with Streptavidin conjugated alkaline phosphatase. Detection with nitro blue tetrazolium chloride/5-Bromo-4-chloro-3-indolyl phosphate (NBT/BCIP) yields a dark blue stain. Nuclei were stained with Nuclear Fast Red Counterstain.

### Statistical analysis

Multiple comparisons were analyzed by ANOVA with Modified Bonferroni post hoc test. A p-value <0.05 was considered significant. For figures, (*) denotes p < 0.05, (**) denotes p < 0.01, and (***) denotes p < 0.001. Cell counts are based on 10 random fields from multiple sections for each condition. Data is represented as the mean (+/−) SEM.

## Abbreviations

ATO: Arsenic trioxide; CISH: Chromogenic *in situ* hybridization; CoTx: Co-treatment with ATO + GCV; EBV: Epstein-Barr virus; GCV: Ganciclovir; NBT/BCIP: Nitro blue tetrazolium chloride/5-Bromo-4-chloro-3-indolyl phosphate; NPC: Nasopharyngeal carcinoma.

## Competing interests

JL and MS in conjunction with Administrators of Tulane Educational Fund have submitted a patent titled “The co-administration of arsenic compounds and anti-herpes virus anti-virals”.

## Authors’ contributions

MDS – Study design, immuno-staining, statistical analysis and drafting of the manuscript; MLS – blind investigator measuring tumor volume; FL – administered injections, immuno-straining; ZL – created CNE1 < BX1 > cell line and study design; EKF – Study design and drafting of the manuscript; JAL - Study design and drafting of the manuscript. All authors read and approved the final manuscript.
